# Establishing a robust preclinical model to investigate early and late radiation-induced skin reactions

**DOI:** 10.1038/s41598-026-39414-6

**Published:** 2026-02-14

**Authors:** P. Ashwini. N. Pai, Kamalesh Dattaram Mumbrekar, Krishna Kishore Mahato, Smitha S Prabhu, Anuradha Calicut Kini Rao, Vijendra Prabhu

**Affiliations:** 1 Manipal Institute of Technology, Manipal Academy of Higher Education, Manipal, India; 2 Department of Radiation Biology and Toxicology, Manipal School of Life Sciences, Manipal Academy of Higher Education, Manipal, India; 3 Department of Biophysics, Manipal School of Life Sciences, Manipal Academy of Higher Education, Manipal, India; 4 Department of Dermatology, Venereology and Leprosy, Kasturba Medical College, Manipal Academy of Higher Education, Manipal, India; 5 Department of Pathology, Kasturba Medical College, Manipal Academy of Higher Education, Manipal, India

**Keywords:** Cancer, Radiation therapy, Radiation-induced skin reactions, Dermatitis, Skin fibrosis, Radiation therapy oncology group, Quality of life, Cell biology, Diseases, Medical research

## Abstract

Radiation-induced skin reactions (RISRs) adversely affect cancer patients by limiting treatment adherence and quality of life (QoL). The current study aimed to establish a murine model for early and late RISR by fractionated radiation, with 30 Gy (10 Gy X 3 sessions) and 50 Gy (10 Gy X 5 sessions) irradiation of the right hind limb of male Swiss albino mice. Early RISRs were monitored for 30 days via blinded RTOG grading, and tissue samples from days 15 and 30 were harvested for histological and morphometric analysis (H&E staining). For late RISR, mice exposed to 50 Gy were examined for 120 days by phenotypical, histological, and morphometric evaluations (H&E and Masson’s trichrome staining). The 30 Gy group presented a median RTOG grade of 2, resolving by day 30, whereas the 50 Gy group presented a median grade of 3, resolving by day 35. Early RISR in the 50 Gy group revealed dermal inflammation, ulceration, cellular infiltration, and reduced hair follicle density, with late effects of dermal indentations, excessive collagen, cellular inflammation, and persistent hair follicle loss (*p* < 0.05). The present murine model effectively replicated early and late RISR clinical and histological features for mechanistic investigations and developing therapeutic strategies in future studies.

**Supplementary Information:**

The online version contains supplementary material available at 10.1038/s41598-026-39414-6.

## Introduction

Radiation therapy is an indispensable technique used for the accurate targeting and treatment of malignant tumors, offering curative or palliative benefits^1^. However, multiple side effects exist due to radiation therapy, such as oral mucositis, xerostomia, nausea, diarrhea, and radiation-induced skin reactions (RISRs), including early dermatitis and late fibrosis of the skin and hyperpigmentation at the target region caused by exposure to ionizing radiation^1–4^. RISRs can be broadly categorized into two main types on the basis of their temporal progression and distinct clinical presentations: early and late RISRs^5^. These are a group of skin reactions that originate from direct cellular harm, leading to inflammatory reactions, ultimately affecting the integrity of the skin barrier and vascular supply around the target region^6,7^.

Clinically, early RISR manifests within one week to three months following initial radiation exposure with varying degrees of severity and symptoms ranging from mild erythema to more severe manifestations, including edema, dry desquamation, moist desquamation, and ulcerations in a few cases^3,8^. Delayed skin reactions in patients lead to late RISR, with complications typically manifesting anywhere from six months to several years post-exposure to radiation^9^. Late RISR manifests as a spectrum of alterations to the skin texture and composition, including chronic ulcerations, soft tissue atrophy, alopecia, stiffness, hyperkeratosis, subcutaneous fibrosis, and radiation-induced telangiectasia^10–12^. Early and late skin reactions affect quality of life and potentially disrupt treatment regimens^13^. Research has demonstrated a clear correlation between the dosage of radiation exposure and the severity of early RISR; with increasing exposure dose, patients experience marked and severe early symptoms^14^. Furthermore, longitudinal studies have indicated that higher radiation doses significantly increase the probability of developing late RISR manifestations, such as fibrosis and telangiectasia, at the irradiated site^15^.

Research conducted on murine models has focused primarily on the progression of early RISR, with less attention given to late RISR^16,17^. Preclinical models, particularly mice, are frequently exploited because of their availability, cost-effectiveness, and capacity to conduct comprehensive mechanistic assessments^18^. However, there is variability regarding the choice of anatomical site for radiation exposure, monitoring duration, total dose, and fractionation scheme due to the lack of a standard protocol^19,20^. Additionally, the emphasis has largely been on administering single doses of radiation, neglecting fractionated dosing, which is more representative of clinical treatment protocols^17,21,22^. Thus, the current study aims to establish a well-characterized, reproducible preclinical model to investigate both short-term and long-term skin reactions to ionizing radiation performed in a single study with fractional irradiation doses of 30 Gy and 50 Gy to the right hind limb of the mice. RTOG (Radiation Therapy Oncology Group) grading, along with semiquantitative and morphometric analyses via H&E staining, was performed to determine early skin reactions. Furthermore, phenotypical observation, semiquantitative scoring metrics, and morphometric analysis with Masson Trichrome staining were performed to establish late RISR conditions within the same murine model.

## Results

RTOG grading revealed early RISR development at the site of irradiation.

**Fig. 1:**
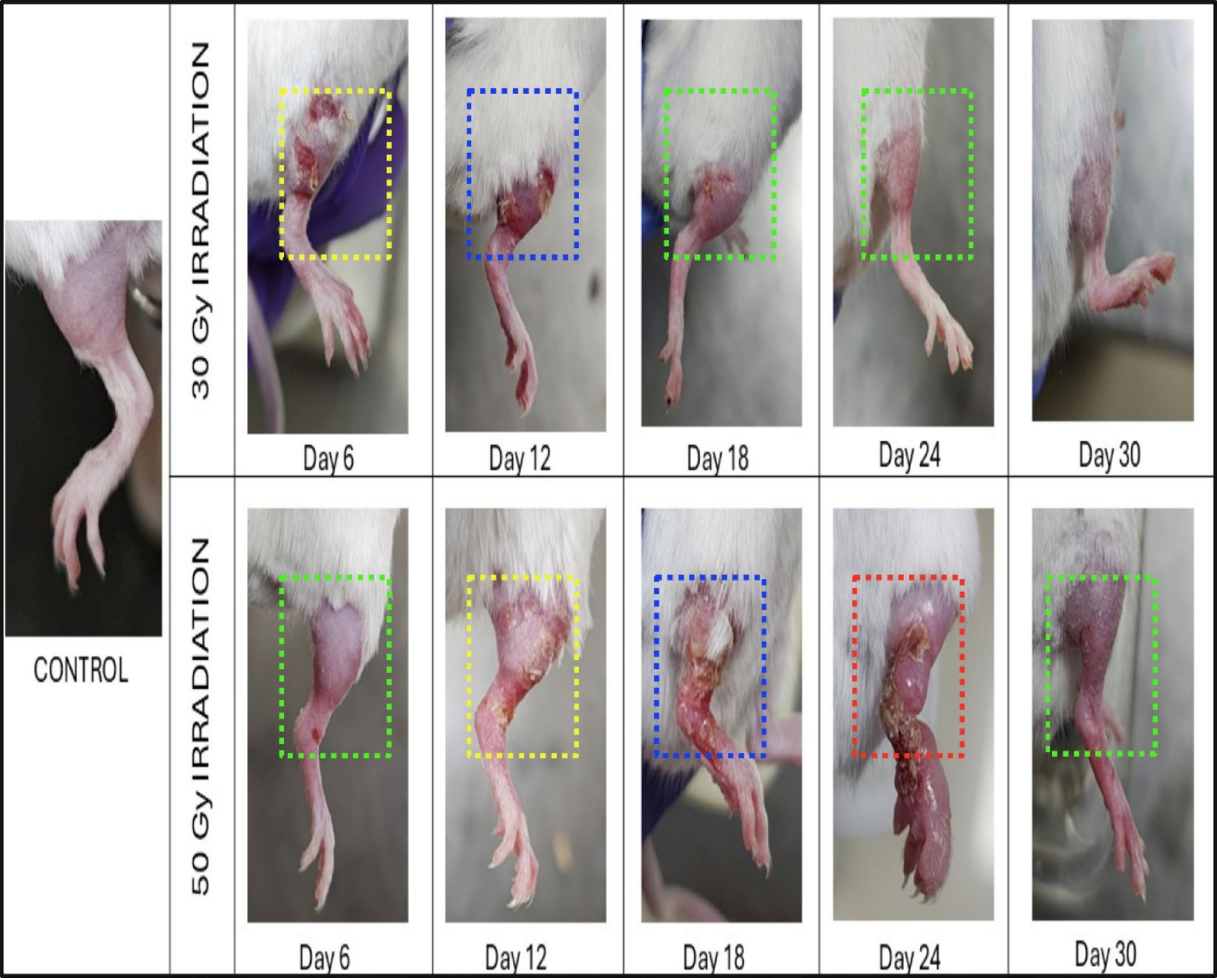
Representative photographic images of the hind limbs of control, 30 Gy- and 50 Gy-exposed mice. The right limb displayed clinical manifestations of early RISR, including erythema (green dotted box), dry and moist desquamation (yellow dotted box), necrosis and ulceration (blue dotted box), and edema (red dotted box)

**Fig. 2:**
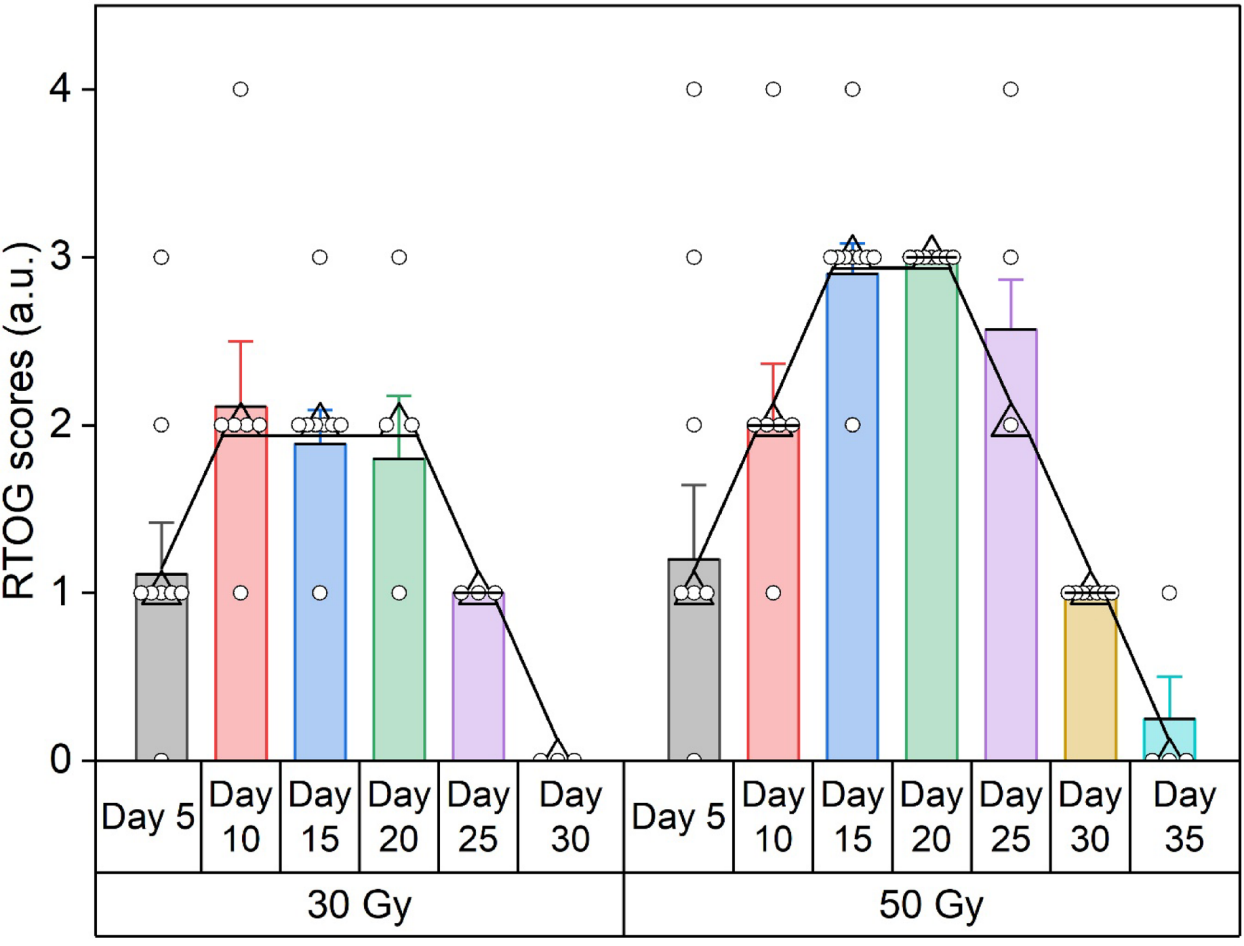
RTOG grade scores of mouse skin irradiated with 30 Gy or 50 Gy of radiation. The data are presented as median scores at each time point

The RTOG grading assessment demonstrated a significant correlation between the exposed radiation dose and the resulting skin damage (Fig. 1). Among the mice exposed to a dose of 30 Gy, 20% presented grade 1, 50% presented grade 2, 10% presented grade 3, and 20% presented grade 4 (Fig. 2). Among the mice exposed to a total dose of 50 Gy, 60% were grade 3, and 40% were grade 4. The assessment of the RTOG scores of the mice revealed that those exposed to 30 Gy of irradiation had a peak median grade of 2 by day 10 post-irradiation, with complete healing and recovery by day 30. In contrast, mice subjected to a higher dose of 50 Gy presented a peak median grade of 3 on day 15 post-irradiation, with a significant number exhibiting grade 4 signs compared with those subjected to 30 Gy of radiation. Moreover, the mice also displayed a noticeable delay in the healing process, with full recovery achieved by day 35.

Morphometric analysis of histological sections revealed early RISR.

**Fig. 3:**
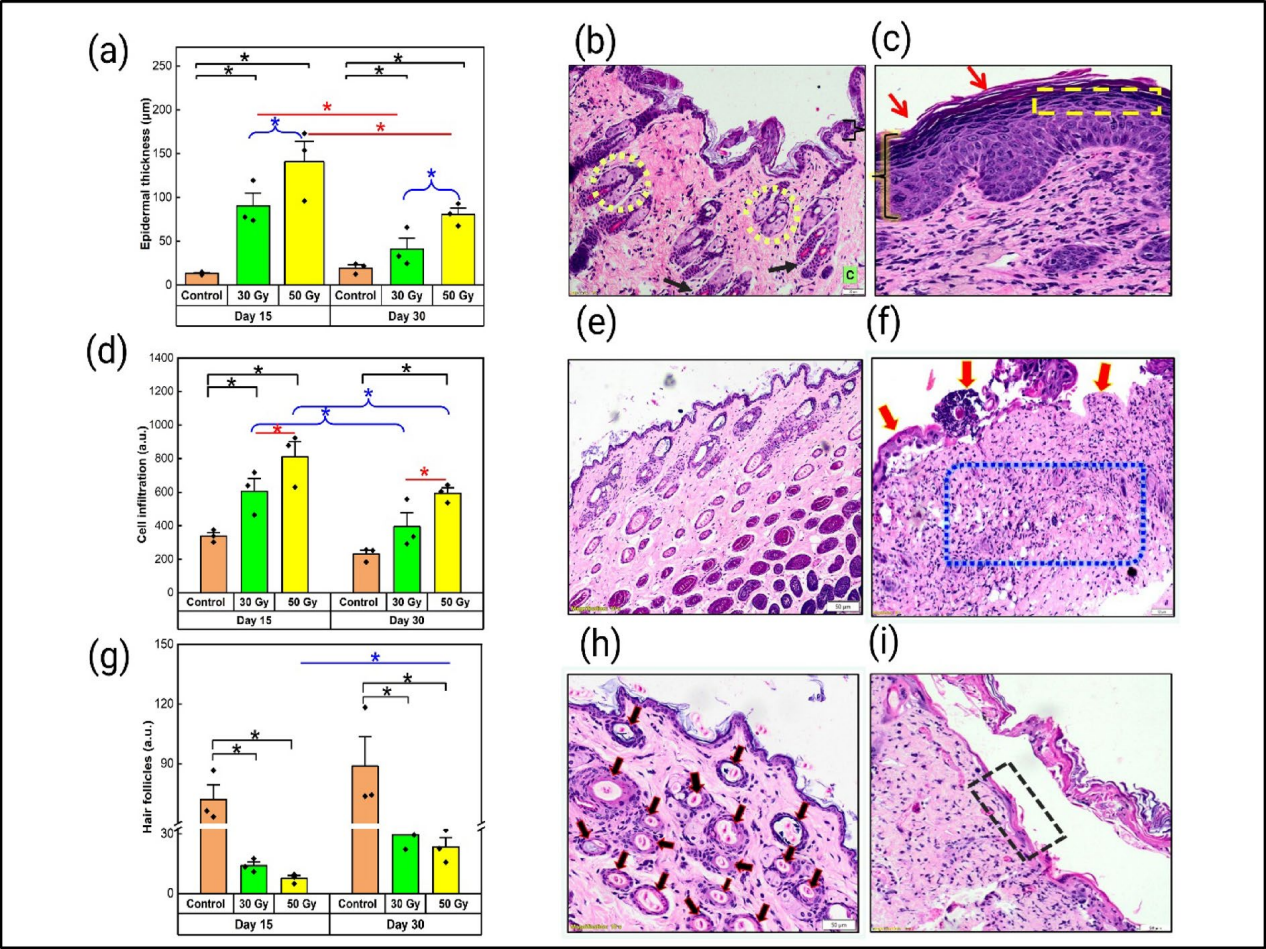
Progression of early RISR indicated by H&E sections and morphometric analysis. (**a**) Morphometric analysis of epidermal thickness. (**b**) Sections of the control tissue with a regular epidermis (flower bracket), sebaceous glands (yellow circle), hair follicles (black arrow), and dermal collagen (C). (**c**) Sections of the irradiated tissue (50 Gy) exhibiting epidermal hyperplasia (flower bracket), hyperkeratosis (red arrow), and hypergranulosis (yellow dotted box). (**d**) Morphometric analysis of cell infiltration. (**e**) Sections of control tissue with normal architecture of the dermis and epidermis. (**f**) Sections of irradiated tissue (50 Gy) with significant dermal inflammation (blue box) and ulceration (red arrow). (**g**) Morphometric analysis of hair follicles. (**h**) Sections of control tissue with abundant hair follicles (black arrow). (**i**) Section of irradiated tissue (50 Gy) exhibiting parakeratosis (black box). The data are expressed as the mean ± SEM. *p < 0.05. (Images III b, III c, III e, III f, III g: 100x total magnification and III i: 200x total magnification)

The epidermal thickness (µm) was significantly greater in the irradiated tissue than in the control tissue, with pronounced thickening observed in the 50 Gy-exposed group (*p* < 0.05) compared with the 30 Gy group on both days 15 and 30. Furthermore, both the 30 Gy and 50 Gy doses had significantly (*p* < 0.05) reduced epidermal thickness on day 30 compared with that on day 15 (Fig. 3a). The evaluation of cell infiltration revealed a significant increase (*p* < 0.05) in the irradiated group on days 15 and 30 for those exposed to 50 Gy compared with the control group, which presented only a minimal baseline level of cells in the dermal region. The group that received 50 Gy of radiation presented notably greater levels of cell infiltration (*p* < 0.05) than did the group that received 30 Gy (Fig. 3 d). Compared with that in the control group, the number of dermal appendages/hair follicles was significantly lower (*p* < 0.05) in the groups exposed to both 30 Gy and 50 Gy on days 15 and 30. On day 15, a substantial reduction in hair follicle number was observed in the group subjected to 50 Gy irradiation (*p* < 0.01) compared with the 30 Gy group (Fig. 3 g).

Semiquantitative assessment of histological sections indicated early RISR in the mice.

H&E-stained sections were assessed and scored for parameters indicating acute skin inflammation and tissue damage, as provided in Table 1. Epidermal hyperplasia was significantly greater on day 15 in the 30 Gy group (*p* < 0.05) than in the 50 Gy group. However, compared with the control group (Fig. 3 b), the 50 Gy-irradiated group presented a marked increase (*p* < 0.01) on day 30 (Fig. 3c). Compared with that in the control group, dermal inflammation in both irradiated groups was considerably greater, and that in the 30 Gy group was significantly greater (*p* < 0.01) on day 15. In contrast, the 50 Gy exposure group also presented increased inflammation (*p* < 0.05) on day 30 (Fig. 3f). Compared with the control group, the 50 Gy treatment group presented pronounced hyperkeratosis and hypergranulosis (*p* < 0.01) on day 30 (Fig. 3 c). Parakeratosis, characterized by retention of nuclei in the stratum corneum due to accelerated keratinization, was observed particularly in the 50 Gy treatment group (Fig. 3i) in comparison with the control (Fig. 3h); however, the quantity of these nuclei was not significant. Compared with those in the control group, no ulcerations were recorded in the 30 Gy-exposed group at either time point (Fig. 3e). Compared with the control and 30 Gy treatment groups, the 50 Gy treatment group presented significant ulceration (*p* < 0.05) on day 15, with complete loss of the epidermal layer (Fig. 3 f). Analysis of hair follicle density revealed a significant decrease (*p* < 0.05) in the group exposed to 50 Gy of radiation (Fig. 3i) compared with the control group on day 30 (Fig. 3h). Additionally, both the 30 Gy- and 50 Gy-exposed groups presented increases in eosinophils and macrophages compared with those of the control; however, these differences were not statistically significant.

**Table 1:** Semiquantitative analysis of parameters from H&E-stained histological sections of irradiated and control tissue collected on days 15 and 30.

Parameters	Control	30 Gy irradiation		50 Gy irradiation	
		Day 15	Day 30	Day 15	Day 30
Epidermal hyperplasia (a.u.)	0	1.67 ± 0.33 ^*^	0.67 ± 0.33	0.67 ± 0.33	3 ± 0 ^**#^
Hyperkeratosis score (a.u.)	0	1.33 ± 0.33	0.67 ± 0.33	0.67 ± 0.33	1 ± 0 ^**^
Hypergranulosis score (a.u.)	0	1 ± 0	0.67 ± 0.67	0.67 ± 0.33	1 ± 0 ^**^
Dermal inflammation (a.u.)	0	2 ± 0 ^**^	1.67 ± 0.33^*^	0.67 ± 0.33	2.33 ± 0.33^*^
Ulceration score (a.u.)	0	0	0	3 ± 0 ^*#^	0
Fibroblast score (a.u.)	0	1 ± 0	0.67 ± 0.67	0.67 ± 0.33	1.67 ± 0.67
Hair follicle at HPF	9.16 ± 0.59	1 ± 0	8.50 ± 3.67	6 ± 0	2 ± 0.81^*^
Eosinophils at HPF	0	8.67 ± 3.78	4.67 ± 3.41	0	4.67 ± 3.41
Macrophages at HPF	0	1.33 ± 1.08	3.33 ± 2.72	0	10 ± 4.71

The data are reported as the mean ± SEM. *p < 0.05; **p < 0.01 compared with the control tissue; ^#^*p* < 0.05 compared with the 30 Gy treatment group.

Phenotypic assessment revealed late RISR in mice.

Among the parameters assessed, a decrease in hair count was noted in the irradiated region from day 40 to day 120 compared with that in the control leg in all four mice evaluated. Additionally, when tactile examination was performed, induration of the skin was identified in the irradiated area on days 90, 100, 110, and 120, indicating the development of mild fibrosis in the mice. Parameters such as edema, telangiectasia, and pigmentation changes were absent in the exposed area. Comprehensive information on the assessed parameters is provided in Supplementary Table S1.

Qualitative assessment of histological samples revealed an accumulation of dermal collagen.

**Fig. 4:**
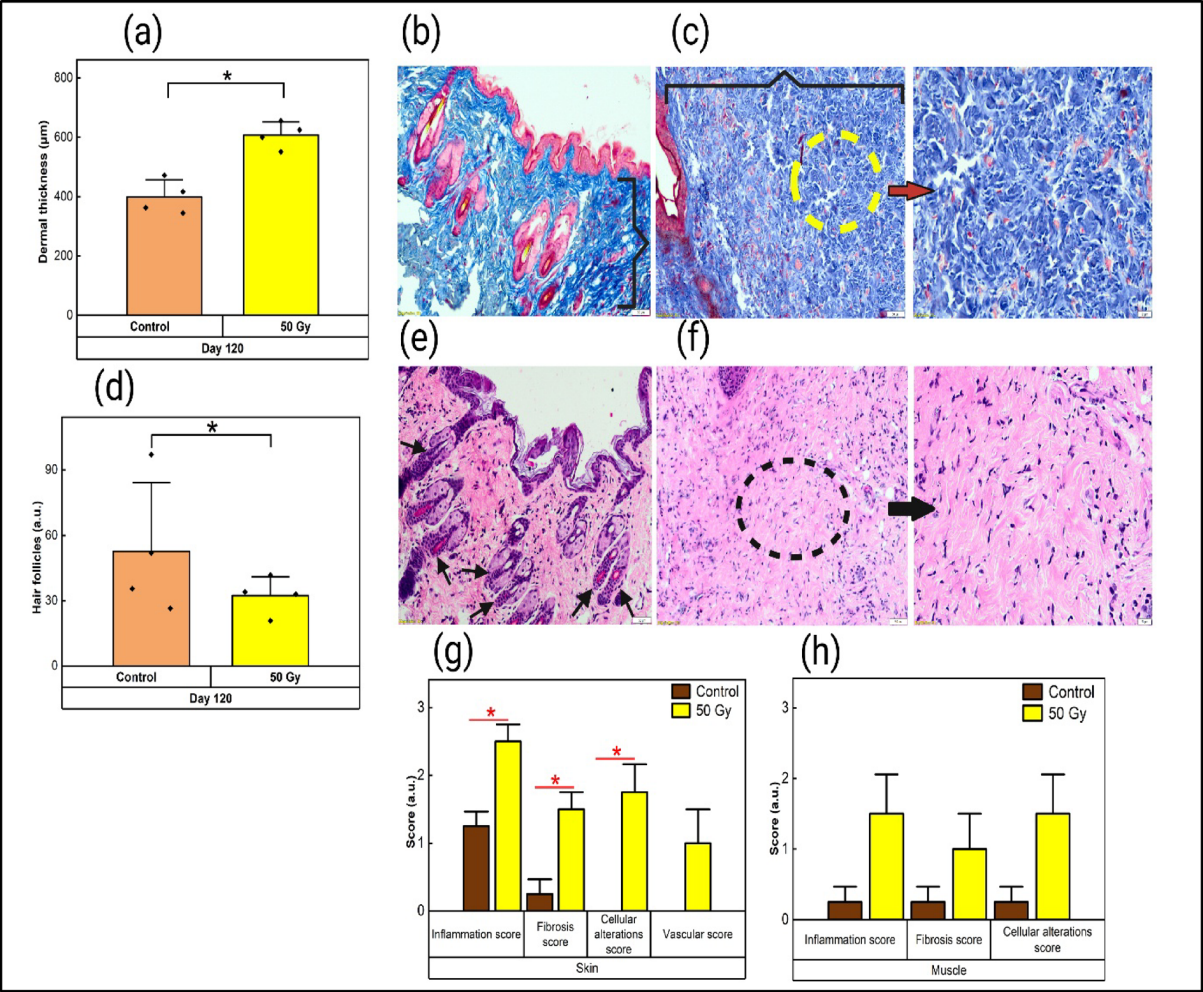
Progression of late RISR depicted by semiquantitative and morphometric analysis of histological sections on day 120 (*n* = 4; 100x total magnification). (**a**) Morphometric analysis of dermal thickness in 50 Gy-irradiated tissue. (**b**) Masson’s trichrome-stained control tissue sections highlighting the regular dermis. (**c**) Masson’s trichrome-stained irradiated tissue Sections (50 Gy) exhibiting increased dermal thickness with disorganized and densely packed dermal collagen (200x total magnification). (**d**) Morphometric analysis of hair follicle density. (**e**) H&E-stained control tissue sections with hair follicles. (**f**) H&E-stained irradiated tissue Sect. (50 Gy) displaying fewer hair follicles with abundant collagen in the dermis (200x total magnification). (**g**,** h**) Semiquantitative scoring of inflammation, fibrosis, cellular alterations, and vascularization in skin and muscle tissue sections from the control and 50 Gy treatment groups. The data are expressed as the mean ± SEM; *p < 0.05. (Images IV b, IV c, IV e, IV f:100x total magnification)

MT staining revealed a densely stained blue area in the dermis (Fig. 4c) characterized by thicker, disorganized, and tightly packed collagen fibers in the dermal region, indicating increased collagen deposition, similar to the characteristic features of fibrosis, compared with the control (Fig. 4b). Additionally, the H&E-stained tissue Sections. (50 Gy) presented a uniform pink hue in the dermis, indicating fibrosis in the dermal region (Fig. 4f) compared with the control tissue (Fig. 4e).

Morphometric and semiquantitative assessment of histological samples indicated late RISR in mice.

Morphometric analysis performed on MT-stained tissue section revealed a significant increase (*p* < 0.05) in the dermal thickness of the irradiated tissue compared with that of the control skin tissue (Fig. 4a). Additionally, a significant decrease (*p* < 0.01) in the number of hair follicles in irradiated skin tissue was observed compared with that in the control skin tissue (Fig. 4d). Semiquantitative assessment was performed on H&E-stained skin and muscle tissue sections. Compared with nonirradiated control tissues, irradiated skin tissue subjected to a cumulative dose of 50 Gy presented a significant increase in parameters such as the inflammation score, fibrosis score, and cellular alteration score (*p* < 0.05), with no significant changes observed in the vascular score (Fig. 4g). Higher scores for inflammation, fibrosis, cellular alterations, and vascularity were recorded in irradiated muscle tissue than in control muscle tissue. However, these differences were not statistically significant (Fig. 4h).

## Discussion

Early and late RISRs are among the most documented side effects that patients encounter during radiation therapy^1^. Such reactions delay the treatment schedule, hindering the effectiveness of the therapy and ultimately affecting patients’ quality of life^13^. There is a lack of a reliable preclinical model that can accurately represent both early and late RISR, hindering clinical translation studies^23^. The development of a preclinical model is essential, as it provides a controlled environment to study the progression of various diseases at different stages, along with the identification of the pathways involved^23^. Preclinical models are also useful in replicating clinical scenarios, allowing the simulation of various disease states and designing interventions that can mitigate tissue toxicity, thereby reducing the risk of adverse effects^24^.

Current studies have indicated that the tissue architecture and cellular organization of the epidermal layers of murine skin are able to critically determine the duration and temporal expression of early and late RISR^25^. Mice exhibit rapid turnover in their skin, making the murine epidermis a hierarchical (H-type) tissue^26^. Early radiation injury causes depletion of stem cells and loss of mature post-mitotic cells, leading to the development of peak erythema and desquamation at approximately 12–20 days, analogous to the 3–6 week acute dermatitis observed in patients who receive breast and chest wall radiotherapy^26,27^. Additionally, severe reactions in H-type tissues can later change to flexible (F-type) tissues, caused by a decrease in stem cells and slow healing, leading to persistent inflammation and fibrogenesis and other late effects (months to years), as observed in clinical cases^27–30^.

Most preclinical investigations have concentrated on the use of high-dose, single-fraction radiation exposure to assess both early and late RISR^12,16,17,23^. Since these studies utilized high-dose, single-fraction radiation exposure, they cannot be directly correlated with clinical settings, as clinical regimens typically involve the use of a fractionated dosing regimen (2 Gy/fraction in conventional treatment), with the cumulative radiation dose ranging between 50 and 60 Gy^31^. At present, few preclinical studies have focused on establishing early or late RISR via fractionated dosing regimens^22,32^. The present study also reported acute skin reactions observed in mice within 15 days of radiation exposure, similar to those reported in prevalent malignancies, such as head and neck radiotherapy^33^ and breast radiotherapy^31^, where similar symptoms have been reported to manifest within 2‒3 weeks of conventional fractionation^34,35^. Hence, the current study focused on selecting a dosing regimen within the clinically implemented treatment dose range to mimic the required clinical manifestations of early and late reactions within a single murine model.

Existing preclinical models that focus on the establishment of early or late skin reactions have other limitations, including the targeted anatomical site. Various anatomical sites have been utilized as targets for radiation exposure, mostly exposing the hindlimb^16,36,37^, dorsal skin^38^, and, in fewer cases, the tail of the murine^17^, the oral cavity, and the salivary glands^21,32,39^, flank skin^40^, and ear pinnae^22^. Anatomical sites such as the tail require modifications to the scoring criteria because of the increased melanin content in that area^17^. Ear pinnae, which are made of cartilage, have a different cellular structure than the rest of the body does^22^. In contrast, the hind limb, which consists of skin, muscle, and bone, has a well-defined anatomical region, with relatively uniform skin tissue composition, isolated from vital systemic organs, easy and precise shielding of the rest of the body, and provides an effective model for studying early skin reactions and the development of fibrosis through long-term monitoring^41,42^.

The RTOG assessment in the present study revealed dose-dependent acute skin damage in the target region, with a greater degree of damage and a delayed healing time for 50 Gy radiation than for 30 Gy radiation, similar to the scoring observed for the monitoring days reported in earlier preclinical and clinical studies^17,31,39^.

In the present study, histological observations, which are considered the gold standard for confirming the development of acute skin reactions, revealed cellular and structural changes at the site of irradiation, including epidermal thickening, hyperkeratosis, and hypergranulosis; reduced hair follicle density in the mice exposed to both 30 Gy and 50 Gy; parakeratosis and ulceration were specifically observed in the group exposed to 50 Gy of radiation, indicating skin damage in a dose-dependent manner, as reported previously^17,38,43^.

Semiquantitative assessment of the histological parameters revealed a significant increase in epidermal thickness in both experimental groups, indicating that the inflammatory response in the skin was related to early RISR development, as described previously^40,43^. Hyperkeratosis observed due to increased proliferation and extreme build-up of cornified keratinocyte cells and hypergranulosis due to dysregulated keratinocyte maturation caused by radiation-induced stress have also been reported previously^17,43^. Significant ulcerations were observed, marked by a complete breakdown of the epidermis with death of keratinocytes and microvascular damage, specifically in the mice exposed to 50 Gy of radiation, which served as a critical clinical endpoint for a severe form of early RISR, as reported in earlier studies^16,17,43^. There was a significant reduction in hair follicle density, which was correlated with inflammatory and epidermal changes in skin tissue exposed to 50 Gy, as reported previously^16,17,44^. There was no significant change observed in the macrophage or eosinophil cell count or the number of fibroblasts in the dermis or epidermis.

The scoring metrics for the group exposed to 50 Gy in the present study also correlated with the RTOG score, highlighting the detrimental effects of higher radiation doses on the structural integrity of skin tissue and the development of associated risks of long-term complications such as chronic ulcers and fibrosis.

Morphometric analysis supported the standard histological analysis assessments performed as objective and reproducible assessments for early RISR establishment. The results of the present study revealed a marked increase in epidermal thickness and dermal cellular infiltration parameters and a substantial decrease in hair follicle density in the tissues exposed to radiation doses of both 30 Gy and 50 Gy compared with those in the control skin tissue, as cited earlier^45,46^. The morphometric analysis in the current study thus further supports earlier findings indicating the successful establishment of an early RISR murine model.

Phenotypic assessments performed in the present study on the 50 Gy exposure group for late RISR via visual inspection of several parameters and tactile examination of skin indentations revealed noticeable development of mild fibrosis, along with a significant reduction in hair growth compared with the contralateral control limb of the same mice, indicating the development of fibrosis within 120 days post-exposure, as reported earlier^13,47,48^. The MT stained skin tissue appeared to have a larger area of blue staining, indicating that the collagen deposition in the dermis with thicker, tightly packed collagen was similar to the development of fibrosis reported previously^12,17,23^.

The semiquantitative scoring for late RISR revealed a significant increase in fibrosis, inflammation, and cellular alterations at the targeted regions compared with those of the control skin tissue. The lower scores given to the muscle tissues suggested that they experienced relatively less injury than did the upper epidermal and dermal layers, as seen in earlier reports^16,17,49^. Morphometric analysis revealed a significant increase in dermal thickness and a notable decrease in hair follicle density in the irradiated tissue, as documented in earlier studies on late RISR^22,32^. Overall, the histological findings, along with the phenotypical assessments, confirmed the development of a fibrotic condition in the mice, indicating the successful establishment of late RISR within the same murine model.

The present preclinical study reported a robust model because of its comprehensive and clinically relevant approach toward the establishment of early and late RISR. The experiment closely resembled the manifestations encountered in clinical radiotherapy, effectively reproducing both the early and late phases of skin reactions experienced by patients. The use of rigorous assessment methods, including RTOG grading and detailed histological analysis, enhanced the objectivity and reliability of the outcomes measured. These advantages have established our preclinical framework as a crucial tool to examine several contributing factors in future studies and to further promote targeted strategies aimed at minimizing toxicity and improving the quality of life of the individuals affected. The present study has certain limitations, including a small sample size and a limited number of time points considered for histological assessments, potentially restricting the ability to obtain detailed insight into the dynamic progression of the observed skin changes.

## Conclusion

In summary, this study successfully established both early and late radiation-induced skin reactions in the same murine model by exposure to cumulative fractionated radiation doses of 30 Gy and 50 Gy. Analysis via RTOG grading, morphometric assessments, and histopathology revealed severe early RISR for 50 Gy exposure. Moreover, late RISR was also established in the same mice previously exposed to 50 Gy, as confirmed by phenotypic examination, as well as histopathological and morphometric assessments. This robust murine model will aid in investigating injury mechanisms and developing targeted therapeutic interventions for radiation medicine in future studies.

## Materials and methods

### Animal care and X-ray radiation

Male Swiss albino mice (weighing 25–30 g; 6–8 weeks old) were obtained and housed at the Central Animal Research Facility, Kasturba Medical College (KMC), Manipal Academy of Higher Education (MAHE), Manipal, India. All the experiments were carried out in accordance with ARRIVE^50^ guidelines and regulations established by the WHO, Switzerland, and the Committee for Control and Supervision of Experiments on Animals (CCSEA), India. The animals were maintained under controlled laboratory conditions at a temperature of 23 ± 2 °C, a relative humidity of 50 ± 5%, and a 12-hour light/dark cycle. Standard laboratory chow and sterile filtered water were provided *ad libitum*. The experimental protocol was reviewed and approved by the institutional animal ethics committee (IAEC), KMC, MAHE, Manipal, India (Approval no: IAEC/KMC/74/2024). For euthanasia, the carbon dioxide inhalation protocol was followed as per the AVMA guidelines for the euthanasia of animals^51^. A total of 20 mice were randomly assigned to two groups of 10 animals each. Group 1 received a cumulative radiation dose of 30 Gy (10 Gy/Session- 3 consecutive days), whereas Group 2 received a cumulative radiation dose of 50 Gy (10 Gy/Session- 5 consecutive days).

**Fig. 5:**
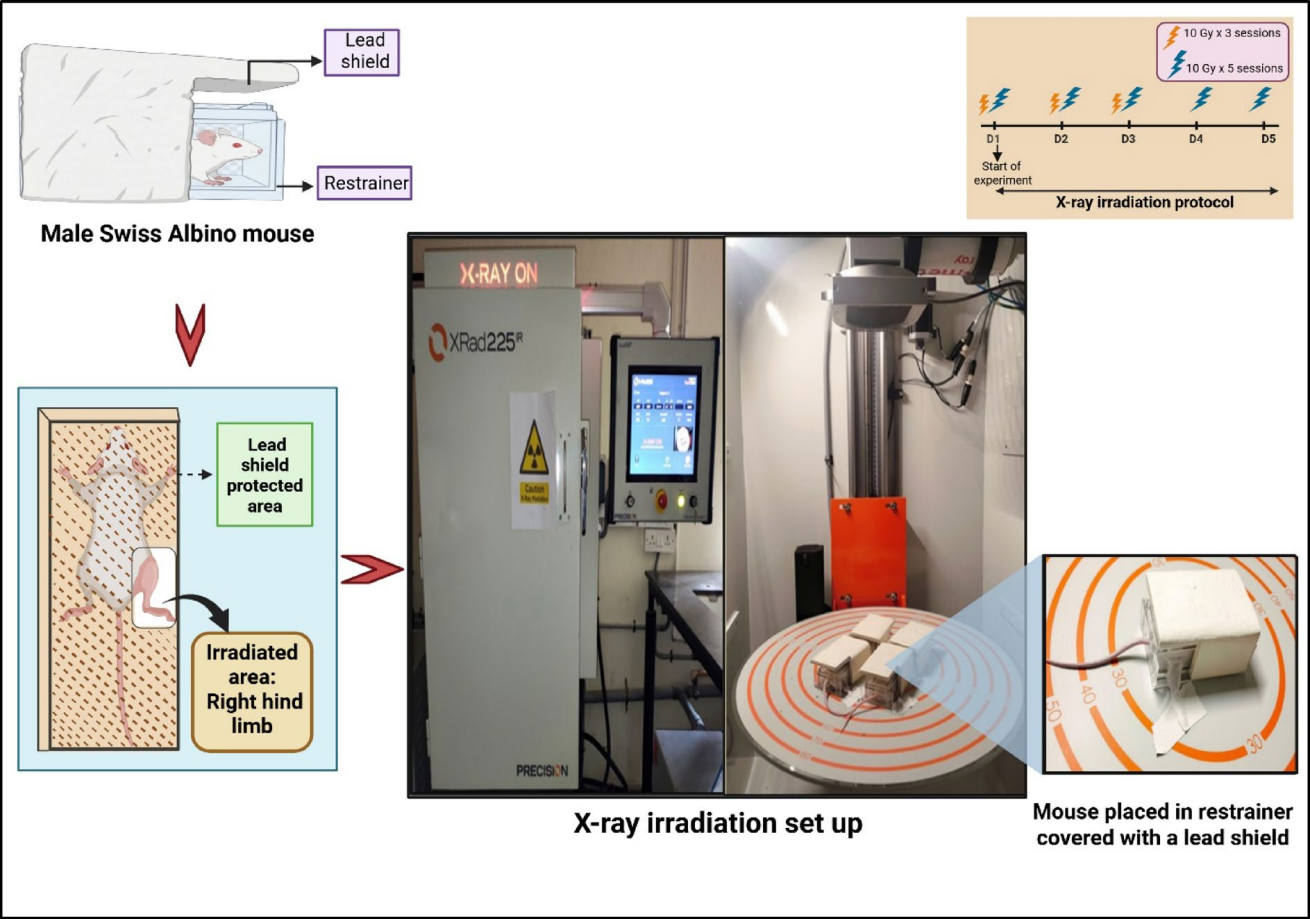
Comprehensive illustration of the fractionated X-ray irradiation experimental setup. A lead shield was used to protect the body of each mouse, exposing only the right hind limb. Images were created via BioRender. https://BioRender.com

**Fig. 6:**
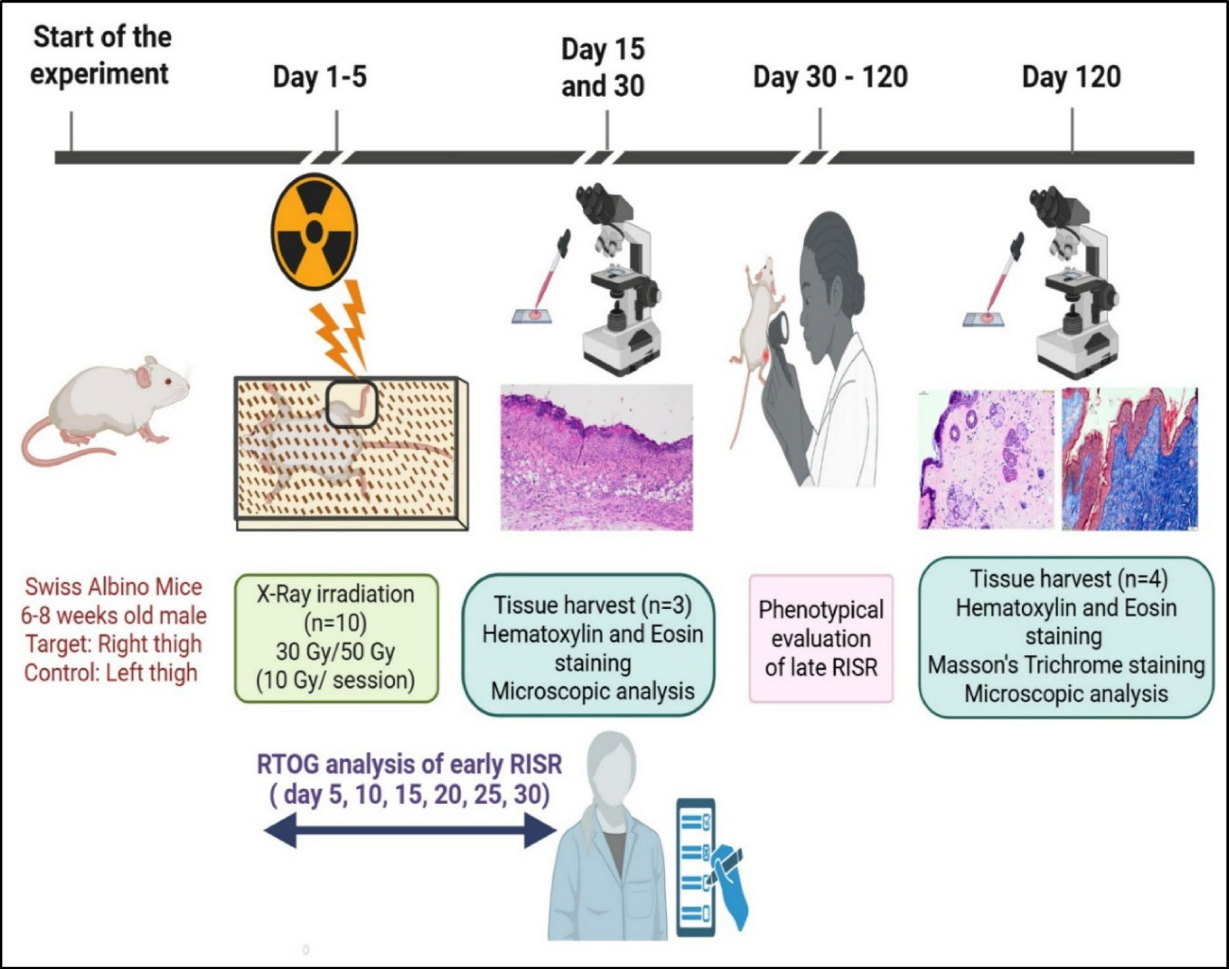
Schematic representation of the experimental timeline indicating the delivery of the radiation session, assessment parameters, and time intervals of tissue sample collection. Images were created via BioRender. https://BioRender.com/812856o

Fractional X-ray irradiation was performed via an X-Rad225-IR X-ray irradiator (Precision X-Ray Inc., USA) system operating at 225 kV, 13 mA tube current, 0.3 mm Cu filter with a dose rate of 84.70 cGy/minute available at the Manipal School of Life Sciences, MAHE, Manipal, India. The mice were exposed to 10 Gy of irradiation per session onto the clean-shaven right thigh, which measured approximately 2 × 2 cm. The clean-shaven left thigh of each mouse served as the control. The mice were positioned in an aerated transparent acrylic restrainer specifically designed for the experiment, avoiding the use of anesthesia during exposure. A lead shield (6 mm in thickness), specifically engineered for this purpose, covered the entire body of each mouse, exposing only the right thigh to X-ray irradiation. A comprehensive illustration is provided in Fig. 5, which shows an X-ray irradiation setup, with the right hind limb positioned outside the restrainer, while the remainder of the body is protected by a lead shield. A detailed experimental timeline for the establishment of both early and late RISR, employed in the current study, is depicted in Fig. 6.

### Assessment of early RISR

#### RTOG grading

Early signs of RISR were monitored for 30 days after radiation to examine the progression of early skin reactions. The mice were physically examined by a practicing dermatologist in a blinded manner on days 5, 10, 15, 20, 25, and 30 and were scored on the basis of the acute radiation morbidity scoring criteria of the RTOG^52^.

#### Histological examination

Skin tissue from both the irradiated and unirradiated areas was collected on days 15 and 30 postirradiation (*n* = 3). The excised tissue was fixed in 10% buffered formalin, processed, and embedded in wax. Five- to seven-micron-thick sections of the tissues were stained with hematoxylin and eosin (H&E) according to the standard protocol. The stained tissue sections were analyzed in a blinded manner by a certified pathologist in 5 fields of view with a bright-field microscope (Olympus CX43, Japan).

Morphometric analysis of the H&E-stained sections was performed by analyzing 3 randomly selected fields of view at different magnifications. Epidermal thickness was measured with a 10x objective (100x total magnification) via Olympus cellSens Imaging software. The vertical line measurement tool was used to measure the distance from the basal layer to the top of the corneal layer of the epidermis. Hair follicle density and infiltrating cell numbers were quantified via ImageJ v1.53t computer image analysis system with a 4x objective (40x total magnification)^17^. Images were obtained from each microscopic field and converted to 8-bit or 16-bit images to evaluate cell infiltration in the dermal region. The free-hand selection tool was employed to delineate and select the region of interest for cell counting, ensuring that edges and boundaries were excluded. With the use of the multipoint tool, each follicle within the RGB image of skin tissue was accurately identified and marked to calculate the average number of hair follicles.

Semiquantitative evaluations were performed for epidermal hyperplasia, hyperkeratosis, dermal inflammation, ulceration, and fibroblast activity. These parameters were graded on a scale from 0 to 3, with a score of 0 representing no or minimal changes, a score of 1 indicating moderate changes, a score of 2 reflecting marked changes, and a score of 3 signifying substantial changes compared with control tissues^38^. Additionally, hair follicle density and immune system components, including eosinophils and macrophages, were quantified in high-power fields (HPFs) with a 40x objective (400x total magnification).

### Assessment of late RISR

#### Phenotypic assessment of late RISR

Delayed skin reactions in the mice were assessed through visual observation by a dermatologist on mice that received a cumulative radiation dose of 50 Gy (*n* = 4). Evaluations were conducted every 10 days, beginning on day 40 and continuing until day 120, to assess the development of subcutaneous fibrosis at the irradiation site, including parameters such as hair loss, pigmentation changes, scarring/fibrosis, telangiectasia, and edema. The parameters were evaluated and documented in a tabular format to ascertain their presence or absence (refer to Supplementary Table S1).

#### Histological examination

Skin, along with muscle tissue from both the irradiated and unirradiated areas, was collected on day 120 post-irradiation (*n* = 4) and processed as described earlier. The tissue Sects. (5–7 μm) were subjected to hematoxylin and eosin (H&E) and Masson’s trichrome (MT) staining^53^. Qualitative analysis was performed on MT-stained tissue sections to specifically detect collagen fibers present within the dermal region of the irradiated skin. H&E-stained sections were utilized for semiquantitative analysis and morphometric analysis of late RISR. Morphometric examination of dermal thickness and hair follicle density was performed as described previously^17^. Semiquantitative scoring by a pathologist included parameters such as fibrosis, vascular changes, inflammatory infiltrates, and cellular reductions and alterations. Each of the parameters was scored using a scale of severity ranging from grade 0 (no change) to grade 3 (severe change) while maintaining a blinded approach^54^.

### Statistical analysis

All the quantitative data were tested for normality. Normally distributed data are expressed as the mean ± standard error of the mean (SEM). The evaluation of significant differences with multiple comparisons was performed with repeated-measures ANOVA, followed by Tukey’s post hoc test where appropriate. Semiquantitative scoring of the histopathological samples was performed via the Mann‒Whitney test. A value of *p* < 0.05 was considered statistically significant.

## Supplementary Information

Below is the link to the electronic supplementary material.

Supplementary Material 1

## Data Availability

All the data generated or analyzed during this study are included in this published article (and its supplementary information files) and will be available upon reasonable request to the corresponding author.
