# Supplementary material for: Establishing a robust preclinical model to investigate early and late radiation-induced skin reactions
Source: Sci Rep. 2026 Feb 14;16:9064. doi: 10.1038/s41598-026-39414-6 (PMC12993074; doi:10.1038/s41598-026-39414-6)
Supplement: Supplementary file 1 — Supplementary Material 1 [file 41598_2026_39414_MOESM1_ESM.docx]

**Establishing a Robust Preclinical Model to Investigate Early and Late Radiation-Induced Skin Reactions**

**Supplementary Table S1**: Phenotypical assessment data for late RISR in mice irradiated with 50 Gy.

| Mice | Visual Parameters | Day 40 | Day 50 | Day 60 | Day 70 | Day 80 | Day 90 | Day 100 | Day 110 | Day 120 |
| --- | --- | --- | --- | --- | --- | --- | --- | --- | --- | --- |
| 1 | Pigmentation change | No | No | No | No | No | No | No | No | No |
|  | Scar/indentation | No | No | No | No | No | No | No | Minimal indentation present | Minimal indentation present |
|  | Edema | No | No | No | No | No | No | No | No | No |
|  | Hair growth | Less compared to the control leg | Less compared to the control leg | Less compared to the control leg | Less compared to the control leg | Less compared to the control leg | Less compared to the control leg | Less compared to the control leg | Less compared to the control leg | Less compared to the control leg |
|  | Telangiectasia | No | No | No | No | No | Present (mild) | No | No | No |
| 2 | Pigmentation change | No | No | No | No | No | No | No | No | No |
|  | Scar/indentation | No | No | No | No | No | No | No | Minimal indentation present | Minimal indentation present |
|  | Edema | No | No | No | No | No | No | No | No | No |
|  | Hair growth | Less compared to the control leg | Less compared to the control leg | Less compared to the control leg | Less compared to the control leg | Less compared to the control leg | Less compared to the control leg | Less compared to the control leg | Less compared to the control leg | Less compared to the control leg |
|  | Telangiectasia | No | No | No | No | No | No | No | No | No |
| 3 | Pigmentation change | No | No | No | No | No | No | No | No | No |
|  | Scar/indentation | No | No | No | No | No | Minimal indentation present | fibrosis present | fibrosis present | fibrosis present |
|  | Edema | No | No | No | No | No | No | No | No | No |
|  | Hair growth | Less compared to the control leg | Less compared to the control leg | Less compared to the control leg | Less compared to the control leg | Less compared to the control leg | Less compared to the control leg | Less compared to the control leg | Less compared to the control leg | Less compared to the control leg |
|  | Telangiectasia | No | No | No | No | No | No | No | No | No |
| 4 | Pigmentation change | No | No | No | No | No | No | No | No | No |
|  | Scar/indentation | No | No | No | No | No | Minimal indentation present | fibrosis present | fibrosis present | fibrosis present |
|  | Edema | No | No | No | No | No | No | No | No | No |
|  | Hair growth | Less compared to the control leg | Less compared to the control leg | Less compared to the control leg | Less compared to the control leg | Less compared to the control leg | Less compared to the control leg | Less compared to the control leg | Less compared to the control leg | Less compared to the control leg |
|  | Telangiectasia | No | No | No | No | No | No | No | No | No |
